# Synovial fibroblasts from children with oligoarticular juvenile idiopathic arthritis induce migration and prolong viability of neutrophils

**DOI:** 10.3389/fped.2024.1376371

**Published:** 2024-07-03

**Authors:** Tobias Schmidt, Anki Mossberg, Elisabet Berthold, Petra Król, Petrus Linge, Anders A. Bengtsson, Fredrik Kahn, Bengt Månsson, Robin Kahn

**Affiliations:** ^1^ Division of Pediatrics, Department of Clinical Sciences Lund, Lund University, Lund, Sweden; ^2^ Wallenberg Center for Molecular Medicine, Lund University, Lund, Sweden; ^3^ Division of Rheumatology, Department of Clinical Sciences Lund, Lund University, Lund, Sweden; ^4^ Division of Infection Medicine, Department of Clinical Sciences Lund, Lund University, Lund, Sweden

**Keywords:** neutrophils, fibroblasts, juvenile idiopathic arthritis, inflammation, rheumatology

## Abstract

**Introduction:**

Little is known of the processes that trigger neutrophil activation in the joint of patients with oligoarticular juvenile idiopathic arthritis (oJIA), and if synovial fibroblasts (S-Fib) play an important role in the activation. Therefore, we aimed to investigate whether S-Fib derived from oJIA patients drive neutrophil activation.

**Methods:**

Synovial fluid (SF) was collected from patients with oJIA. S-Fib were isolated from the SF of *n* = 7 patients through passaging. Subsequently, the S-Fib were primed or not with 20% of pooled SF. Supernatants were used to study migration of neutrophils in a transwell system. Additionally, the influence of S-Fib on neutrophils were studied in co-cultures. Phenotype and viability were assessed by flow cytometry. Neutrophil function was tested through the production of reactive oxygen species (ROS), and supernatants were tested for myeloperoxidase (MPO) release and elastase activity.

**Results:**

Supernatants of S-Fib induced neutrophil migration (*n* = 5, *p* = 0.0491), which was further pronounced using supernatants from SF-primed S-Fib (*p* = 0.0063). Additionally, co-culture between SF-primed S-Fib and neutrophils resulted in prolonged viability (*n* = 5, *p* = 0.0094), with little effect on activation markers, e.g., CD11b. Conversely, co-culture did not induce functional alterations (*n* = 4), such as production of ROS (*p* > 0.1570), release of MPO (*p* > 0.4934) or elastase activity (*p* > 0.0904). Finally, supernatant stimulation did not replicate the results of prolonged viability (*p* = 0.9102), suggesting a role of cell-contact.

**Conclusion:**

S-Fib from patients with oJIA induce migration of neutrophils via soluble mediators and, in addition, S-Fib prolong neutrophil viability in a cell-contact dependent manner. These mechanisms could be important for accumulation of neutrophils during arthritis.

## 1 Introduction

Juvenile idiopathic arthritis (JIA) is a heterogenous inflammatory joint disease in children with partially unknown etiology and pathogenesis (1). The most common subgroup in the western world is oligoarticular JIA (oJIA), which is also the subgroup believed to be most unique for children (2). JIA is thought to develop from a combination of genetic- and environmental factors, resulting in a dysfunctional immune system and a breach in immune tolerance. The contribution of lymphocytes in the pathogenesis has been extensively explored, and studies have identified clonally expanded inflammatory *T*-cells in the joint (3). However, the role of myeloid cells, including that of our most common immune cell, the neutrophil, has been less explored.

We and others have previously shown that synovial neutrophils have an activated phenotype in oJIA (4, 5). For example, they express a higher degree of activation markers, such as CD11b and CD66b, compared to circulating neutrophils (4). In addition, they also express atypical markers to neutrophils, such as HLA-DR, suggesting a role in antigen presentation (4, 6). Functionally, synovial neutrophils have reduced phagocytosis, supporting reduced clearance (4). Moreover, synovial neutrophils fail to suppress *T*-cell proliferation, a feature of circulating neutrophils (7). Mechanistically, these alterations are partly driven by migration and reactive oxygen species (ROS) (7). Finally, Grieshaber-Bouyer et al. suggest that the synovial neutrophil phenotype could partly be induced *in vitro* by interferon-*γ* stimulation and prolonged culture (6). However, the precise mechanisms of how synovial neutrophils acquire their functional alterations remains to be determined.

Synovial fibroblasts (S-Fib) are gaining increasing attention as drivers of inflammation in arthritic diseases [such as rheumatoid arthritis (RA)], e.g., as activators of immune cells (8). For example, S-Fib drive metabolic alterations in macrophages, resulting in increased viability, whilst macrophages promote fibroblast invasiveness (9, 10). We have recently shown that S-Fib from adults drive inflammatory monocyte activation (11). However, there are fewer studies on the role of S-Fib in JIA, but one study suggest that *T*-cell derived cytokines drive S-Fib activation, resulting in increased leucocyte attachment (12). Taken together, these studies highlight the interplay between S-Fib and immune cells as an important factor in arthritis. Thus, interactions with S-Fib represent a potential source of the synovial neutrophil phenotype.

Indeed, in RA, studies suggest that S-Fib interact with neutrophils. For example, neutrophil-derived proteins increased S-Fib attachment to cartilage (13). On the other hand, S-Fib likely have an important role in the recruitment of neutrophils through crosstalk with endothelial cells (14). Additionally, one study in RA suggest that NETs can be taken up and presented by S-Fib, promoting autoreactivity (15). Finally, S-Fib from RA patients promote neutrophil viability (16). However, to our knowledge, there are no studies looking into the role of S-Fib in driving neutrophil activation in oJIA. Given that neutrophils in oJIA show an activated phenotype with functional alterations, it is crucial to determine how the neutrophils acquire this phenotype. Since S-Fib have a role in driving activation of other immune cells, we speculate that they may also be involved in inducing neutrophil migration and subsequent phenotype. Thus, the aim of this study was to characterize if S-Fib from oJIA patients induced activation and drive functional- and phenotypic alterations in healthy neutrophils.

## 2 Method

### 2.1 Patient material and clinical characteristics

This study enrolled *n* = 15 patients diagnosed with oligoarticular juvenile idiopathic arthritis (oJIA) based on the criteria set by the International League of Associations for Rheumatology (ILAR). The participants were recruited from the Department of Pediatric Rheumatology at Skåne University Hospital in Sweden between 2016 and 2023, whilst undergoing therapeutic joint aspiration. Patients were included upon written informed consent from the patients and/or their legal guardians/next of kin. Ethical approval for the study was granted by the Regional Ethical Review Board for southern Sweden (2016/128).

Synovial fibroblasts (S-Fib) were isolated from the synovial fluid (SF) of seven patients, while SF for priming experiments was pooled in equal proportions from a minimum of 8 patients with oJIA. Some patients were used only for S-Fib isolation, some for isolation of SF for pooling and some for both. The process of isolating cells and SF is detailed below. Clinical characteristics of the patients involved in the study are described in Table 1. Notably, patients were either newly diagnosed or in inactive disease without receiving disease-modifying anti-rheumatic drugs (DMARDs) or oral/intra-articular steroids for the past six months preceding inclusion, with the exception of one patient who had been administered methotrexate three months before sampling. All patients contributing with S-Fib had been treated with non-steroidal anti-inflammatory drugs (NSAIDs), but no other drugs. In addition, half of the patients donating SF for priming experiments received NSAIDs, while the other half was untreated at sampling.

**Table 1:** Patient characteristics.

Patient	Sample type	oJIA type	Sex (m/f)	Treatment	ANA	Anti-CCP	RF	^§^Disease duration (months)	^#^Age (years)
1	S-Fib	pers	m	NSAID	Pos	X*	X*	2	8.5
2	S-Fib	ext	f	NSAID	Pos	Neg	Neg	0	2
3	S-Fib	pers	f	NSAID	Pos	NA	NA	10	3.5
4	S-Fib, SF	pers	f	NSAID	Pos	NA	NA	108	11
5	S-Fib	ext	f	^+^MTX, NSAID	Pos	Neg	Neg	63	8
6	S-Fib, SF	ext	m	NSAID	Pos	X*	X*	8	6.5
7	S-Fib	pers	f	NSAID	Pos	Neg	Neg	0	2
8	SF	pers	m	0	Pos	Neg	Neg	139	15
9	SF	pers	f	0	Pos	Neg	Neg	168	16.5
10	SF	pers	m	NSAID	Pos	X*	X*	103	12.5
11	SF	pers	m	0	Neg	Neg	Neg	8	15
12	SF	ext	f	0	Pos	Neg	Neg	35	7.5
13	SF	pers	f	NSAID	Pos	Neg	Neg	0	11.5
14	SF	pers	f	NSAID	Pos	X*	X*	0	4
15	SF	pers	f	0	Pos	Neg	Neg	43	5

Clinical and laboratory data of the 15 patients from which synovial fibroblasts and/or the synovial fluid were collected from.

^§^ Disease duration at time of sampling, ^#^Age at time of sampling, X*—sample not analyzed, ^+^MTX off treatment (methotrexate) for 3 months prior to inclusion, pers, persistent oJIA; ext, extended oJIA; ANA, anti-nuclear antibodies; NSAID, non-steroidal anti-inflammatory drugs; CCP, cyclic citrullinated peptide; RF, rheumatoid factor; SF, synovial fluid; S-Fib, synovial fibroblasts.

### 2.2 Isolation and culture of synovial fibroblasts

Healthy S-Fib were purchased from Cell Applications and maintained in fibroblast-like synoviocytes (FLS) growth medium (Cell Applications). For patient S-Fib isolation, synovial fluid (SF) underwent centrifugation at 500 g for 10 min. The resulting cell pellet was washed twice with PBS and then placed in FLS medium in T-25 flasks (Sarstedt). A second centrifugation of the SF was performed at 800 g for 10 min, and the supernatant was frozen at −80°C for future use. The initially seeded cells were washed with fresh medium 24–48 h later to discard non-adherent cells. Subsequently, the cells were passaged approximately three times until S-Fib-like cells became the predominant cell type, as previously reported (17–19). The identity of the S-Fib was confirmed by flow cytometry for the expression of fibroblast markers, such as Cadherin-11 and CD90. The cells were then frozen at −80°C until needed, with usage limited to passage 10.

For co-culture experiments and priming, S-Fib cells were detached, and 2,000 cells/well were seeded in 96-well plates (Falcon) in FLS growth medium. The S-Fib were allowed to rest for 24 h, followed by either priming or no priming using FLS growth medium supplemented with 20% of pooled SF for an additional 48 h.

### 2.3 Neutrophil isolation and co-culture

Granulocytes were isolated from heparinized whole blood from adult healthy controls through density centrifugation (lymphoprep) at 620 g for 20 min with low break. The upper layer were discarded and the lower fraction containing granulocytes (mainly neutrophils) and red blood cells (RBCs) was used. RBCs were sedimented using 1.5% dextran T500 (Pharmacosmos) in saline solution for 20 min, RT. The cell rich supernatant was collected, centrifuged and remaining RBCs were lysed by 20–25 s incubation with sterile H_2_O, followed by restoration of isotonicity with 1:1 ratio of 1.8% saline. The cells were subsequently centrifuged, resuspended in RPMI-1640 with 10% normal human serum (NHS), counted (XN-350, Sysmex) and adjusted to 1 × 10^6 ^/ml. S-Fib (cultured as described above) were washed twice with RPMI-1640 medium before the addition of 10^5^ neutrophils. Neutrophils alone served as control. The cells were cultured for 24 h and analyzed as described below.

### 2.4 Migration assay

To generate S-Fib conditioned supernatants, S-Fib cells were initially seeded at a density of 10^4^ cells/well in a 24-well Falcon plate using FLS growth medium. After 72 h of cultivation, 20% of pooled SF was either added or omitted, and the S-Fib were subsequently cultured for 48 h (priming). Following this, the cells underwent two washes with RPMI-1640 medium, and serum-free RPMI-1640 was added, with the cells being cultured for an additional 24 h before harvesting the supernatants.

For the migration assay, a 24-well plate with 5 µm pore-sized transwell inserts (Corning) was used. Briefly, HMEC endothelial cells (ATCC) were seeded in the wells and allowed to reach confluence in MCDB-131 medium supplemented with 10% fetal bovine serum, 10 ng/ml hEGF, non-essential amino acids, L-glutamine (1 mM), sodium pyruvate, penicillin, and streptomycin (PenStrep). Prepared inserts were placed in a new 24-well plate containing S-Fib supernatants pooled from four donors. A negative control with RPMI-1640 medium alone was included. Neutrophils, isolated as previously outlined and adjusted to 2 × 10^6 ^/ml in serum-free RPMI-1640, were added (100 µl) to the top of the inserts. The plate was then transferred to an incubator (37°C, 5% CO_2_), allowing migration to occur for 80 min. Subsequently, the inserts were discarded, and cells at the bottom of the wells were harvested by gentle pipetting in PBS/1 mM EDTA. Neutrophils were incubated with a 1:200 dilution of anti-CD66b (clone: G10F5, Alexa Fluor 647, BD) for 20 min at RT. Finally, the cells were washed with PBS, resuspended in 100 µl, and subjected to analysis using flow cytometry (CytoFLEX). The number of CD66b^+^ neutrophils/µl was used for subsequent analysis.

### 2.5 Surface marker and apoptosis analysis of neutrophils

Following co-culture with S-Fib as described above, neutrophils were detached through gentle pipetting and one wash of the wells using PBS/1 mM EDTA, and centrifuged. Next, they were stained with anti-CD66b (1:50, clone: G10F5, FITC, BD) and anti-CD11b (1:200, clone: ICRF44, Alexa fluor 700, BD). The cells were incubated for 25 min, RT. Next, the cells were washed with PBS and resuspended in Annexin V binding buffer (BD biosciences) and stained with 1:200 Annexin V (FITC, BD) for 15 min, followed by analysis using flow cytometry (CytoFLEX).

In some experiments, supernatants were collected from S-Fib cultured for 24 h in 96-well plates (Falcon) in RMI-1640 with 10% NHS. These were then used at 1:1 ratio with fresh medium to stimulate freshly isolated neutrophils for 24 h, followed by analysis of apoptosis (as described above).

### 2.6 Elastase activity

Supernatants were collected following S-Fib-neutrophil co-culture and stored at −80°C until use. An elastase activity assay (EnzChek) was performed according to the manufacturer's instructions. The plate was read at 485/535 nm and analysed using the Wallac 1420 software (version 3.0, PerkinElmer Life Sciences) and Microsoft Excel.

### 2.7 ROS production

Following co-culture between S-Fib and neutrophils, as described above, the neutrophils were detached using gentle pipetting and the wells were washed once with PBS/1 mM EDTA. The cells were subsequently washed once with PBS and resuspended in 100 µl RPMI-1640 supplemented with 5% NHS. The cells were transferred to a black 96-well plate (Thermo Fisher) and placed in a 37°C pre-heated plate reader (VICTOR^3^, 1420 Multilabel Counter, PerkinElmer Life Sciences) for 10 min. Next, 10 µM H_2_DCFDA was added to each well, and reactive oxygen species (ROS) production was induced by stimulation using 10 ng/ml PMA. Unstimulated cells and unstained cells served as controls. The plate was analysed at different time points up to 1 h of incubation. The plate was read at 485/535 nm and analysed using the Wallac 1420 software (version 3.0, PerkinElmer Life Sciences) and Microsoft Excel.

### 2.8 MPO ELISA

Following co-culture between S-Fib and neutrophils, supernatants were collected and frozen in −80°C until use for MPO analysis. The samples were analyzed using the LSBio Human MPO ELISA kit (LifeSpan BioSciences) according to the manufacturer's instructions. Supernatants were diluted 1:30. The plate was read at 450 nm and was analyzed using the Wallac 1420 software (version 3.0, PerkinElmer Life Sciences) and Microsoft Excel.

### 2.9 Statistics

The analysis of flow cytometry data was performed using the CytExpert software (version 2.3) or Kaluza (version 2.1, both from Beckman Coulter). Presentation of data includes the mean with standard deviation unless specified otherwise. Comparison of fold change (FC) data was performed by one-sample t-test and the hypothetical mean of 1. Comparison of data among three groups was performed through repeated measures-one-way ANOVA, followed by Tukey's multiple comparisons test. GraphPad Prism 9 and Microsoft Excel were used for data analysis.

## 3 Results

### 3.1 Synovial fibroblasts from patients with oligoarticular juvenile idiopathic arthritis induce neutrophil migration

**Figure 1:**
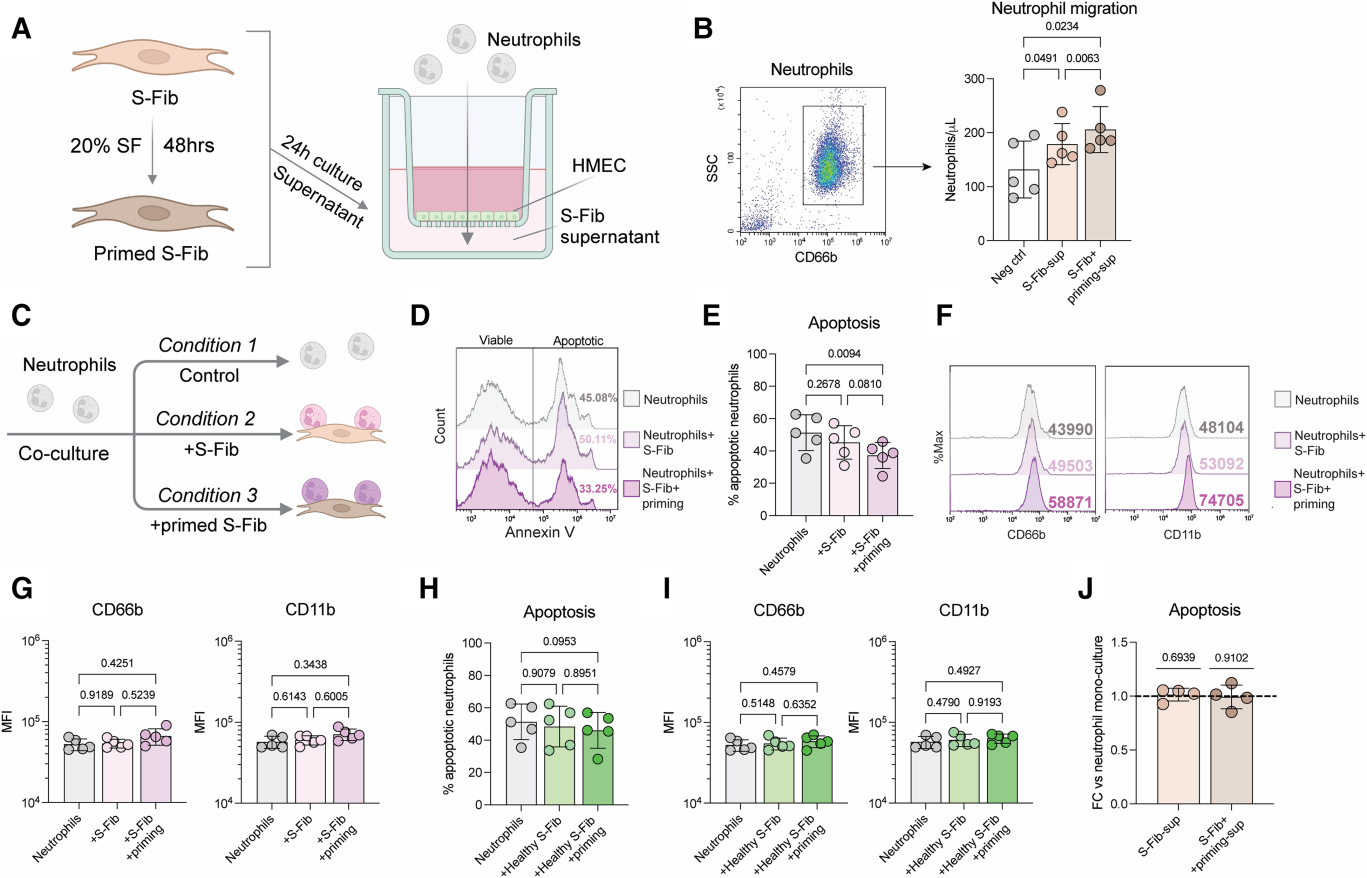
Synovial fibroblasts drive neutrophil recruitment and prolong their viability, with limited effect on activation markers. (**A**) Schematic illustration of how supernatants from four donors of oJIA synovial fibroblasts (S-Fib), with or without prior priming for 48hrs with a pool of synovial fluid (SF), were used to induce migration of healthy neutrophils in a transwell system seeded with endothelial cells (HMEC). (**B**) Neutrophils in the lower chamber were identified based on CD66b expression and counted using flow cytometry. (**C**) Overview of how neutrophils were co-cultured with S-Fib for 24 h, with or without prior priming of the S-Fib. They were subsequently analyzed by flow cytometry for apoptosis and surface marker expression (**D**) representative histograms of the degree of apoptosis following flow cytometry using annexin V staining. (**E**) level of apoptosis as percentage annexin V positive cells in *n* = 5 neutrophil donors. (**F**) the expression of two surface markers in neutrophils following co-culture as representative histograms following flow cytometry analysis. (**G**) the expression as MFI values in *n* = 5 neutrophil donors. In parallel, healthy S-Fib (*n* = 3) were also used as a negative control to study (**H**) the degree of apoptosis in *n* = 5 neutrophil donors and (**I**) the expression of activation markers. (**J**) Supernatants from S-Fib cultures were used to activate neutrophils at a 1:1 ratio for 24 h. Viability was measured using Annexin V, and data represents the fold change (FC) to the control (neutrophils cultured in medium alone). Each data point represents a unique neutrophil donor, which in turn is the average of co-culture with 3–5 different donors of S-Fib. Data is presented as mean ± standard deviation. Statistics was performed using repeated measures-one-way ANOVA with Tukey's multiple comparisons test, except for (**J**), which was performed using one-way t-test and the hypothetical mean of 1. S-Fib, synovial fibroblasts; oJIA, oligoarticular juvenile idiopathic arthritis; SF, synovial fluid; MFI, median fluorescence intensity. Figures 1A,B were created using BioRender.com.

We hypothesized that the inflammatory environment could be important for the phenotype of synovial fibroblasts (S-Fib) in oligoarticular juvenile idiopathic arthritis (oJIA). Thus, we primed the S-Fib with 20% of pooled synovial fluid (SF) from oJIA. Subsequently, we collected supernatants to investigate if these cells could be responsible for neutrophil recruitment. In a transwell system (Figure 1A), supernatants from cultured oJIA S-Fib were able to induce migration of healthy neutrophils (*n* = 5, *p* = 0.0491), which was further pronounced using supernatants from SF-primed S-Fib (*n* = 5, *p* = 0.0063, Figure 1B). Therefore, these findings could be compatible with that S-Fib are able to recruit neutrophils from the circulation, especially following exposure to the arthritic environment of the joint.

### 3.2 Synovial fibroblasts prolong neutrophil survival without inducing activation markers

As S-Fib drive neutrophil migration, we aimed to explore if they promote activation. Healthy neutrophils were thus co-cultured with S-Fib or SF-primed S-Fib from oJIA patients (Figure 1C). As a negative control, we also included healthy S-Fib in these experiments. Co-culture between SF-primed oJIA S-Fib and neutrophils (*n* = 5) prevented neutrophil apoptosis (Figures 1D,E, *p* = 0.0094), as measured by the percentage of annexin V positive neutrophils. A similar trend, although not significant, was observed in co-cultures with unprimed S-Fib (*p* = 0.2678). Furthermore, at the surface marker level, there were no significant changes in expression of either CD66b or CD11b on neutrophils (*n* = 5) following co-culture, although there was a trend of higher expression in neutrophils co-cultured with SF-primed S-Fib (Figures 1F,G). The effect of healthy S-Fib om neutrophils followed a similar trend but did not significantly affect apoptosis (Figure 1H) or the expression of activation markers (Figure 1I). Thus, co-culture with primed oJIA S-Fib results in prolonged viability, but without inducing expression of activation markers, in healthy neutrophils. Finally, the effect of healthy S-Fib on neutrophils was minor.

### 3.3 The prolongation of neutrophil viability is not due to soluble factors

As co-culture with oJIA S-Fib prolonged neutrophil viability, we set out to investigate if this effect was due to soluble factors. Supernatants were collected from S-Fib cultures and used to stimulate neutrophils for 24 h. Compared to medium alone, S-Fib supernatants did not prolong neutrophil viability (*n* = 4), regardless of whether the supernatants originated from unprimed S-Fib (*p* = 0.6939) or primed S-Fib (*p* = 0.9102) (Figure 1J). Thus, direct cell-cell interactions are likely important in prolonging neutrophil viability.

### 3.4 Synovial fibroblasts do not induce functional alterations in healthy neutrophils

**Figure 2:**
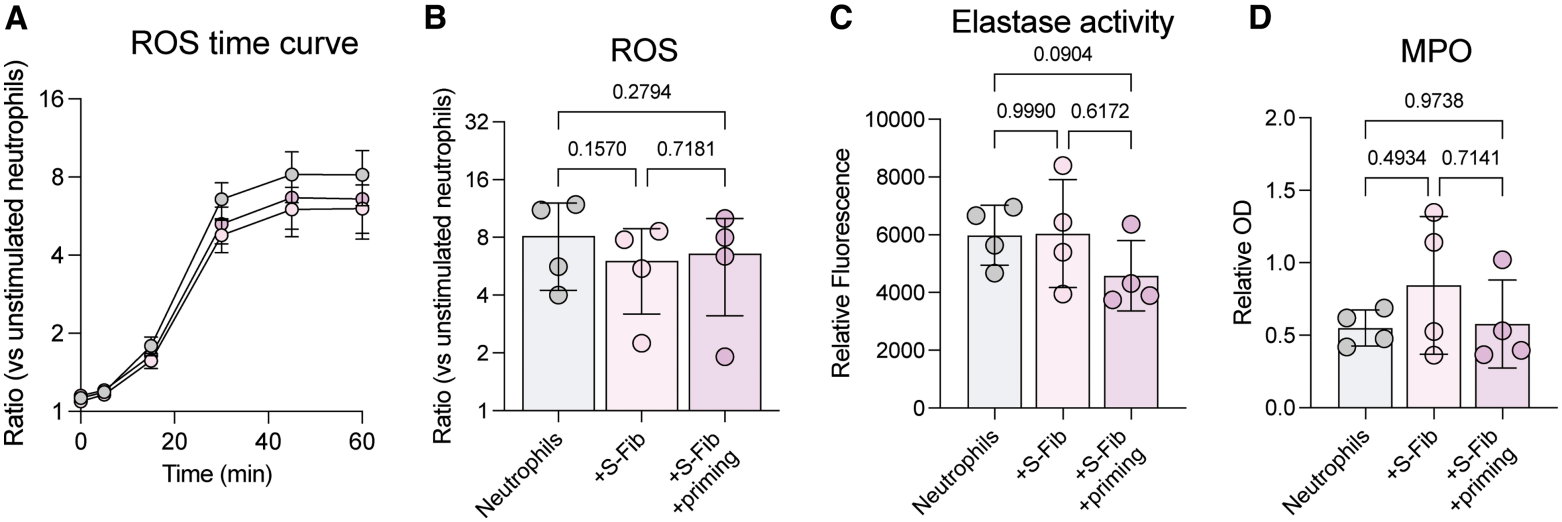
The effects of synovial fibroblast co-culture on neutrophil function are minor. Neutrophils were investigated for functional alterations following co-culture for 24 h with synovial fibroblasts (S-Fib). (**A**) Reactive oxygen species (ROS) was measured in *n* = 4 neutrophil donors following H_2_DCFDA staining and PMA activation, over different time points, or (**B**) at 60 min. Data is presented as fold change/ratio vs. unstimulated control. (**C**) Elastase activity or (**D**) MPO levels were measured in supernatants following co-culture (*n* = 4). Each data point represents a unique neutrophil donor, which in turn corresponds to the average of co-culture with *n* = 5 donors of S-Fib. Data were analyzed using repeated measures-one-way ANOVA with Tukey's multiple comparisons test. S-Fib, synovial fibroblasts; ROS, reactive oxygen species; PMA, phorbol 12-myristate 13-acetate; OD, optical density, MPO, myeloperoxidase.

Co-culture between healthy S-Fib and neutrophils did not influence the neutrophil viability or phenotype, whilst oJIA S-Fib prolonged neutrophil viability but had little effect on activation markers. Thus, as oJIA S-Fib did display some influence on neutrophils, we set out to investigate further whether neutrophils are functionally affected following co-culture with oJIA S-Fib. First, we stimulated neutrophils with PMA following co-culture and measured reactive oxygen species (ROS) production (Figures 2A,B). There was no statistical difference between the groups, although co-cultured neutrophils tended to have decreased ROS production compared to neutrophils alone (*n* = 4). A similar observation was made analyzing elastase activity in supernatants (Figure 2C), were there was no statistical difference, but a trend towards lower activity in supernatants from co-culture with SF-primed S-Fib. Finally, we analyzed MPO levels in supernatants (*n* = 4), and again, we found no difference between the conditions (Figure 2D). Hence, the effect of co-culture between S-Fib and neutrophils are minor at the functional level, with a trend towards lower activity in co-cultures with SF-primed S-Fib, potentially reflecting the increased viability in this setting.

## 4 Discussion

Neutrophils gain increasing attention as drivers of synovial inflammation in patients with oligoarticular Juvenile Idiopathic Arthritis (oJIA). However, the mechanisms driving neutrophil activation in the joint remain elusive. Here, we set out to investigate the role of synovial fibroblasts (S-Fib) in driving neutrophil recruitment and activation. Our results highlight that S-Fib promote neutrophil migration, especially upon prior priming with inflammatory synovial fluid (SF). Additionally, SF-primed S-Fib prolong neutrophil survival. Still, S-Fib had limited effect on the neutrophil phenotype and function, such as the production of reactive oxygen species (ROS) and degranulation. These results highlight a role of the inflammatory environment in driving activation of S-Fib which, in turn, recruit and prolong viability of neutrophils. Yet, the influence of S-Fib on the phenotype and function of neutrophils is minor, suggesting that other processes drive synovial neutrophil activation.

Neutrophils are believed to drive inflammation in arthritis, as they display phenotypical alterations and have altered effector functions. For example, studies in both JIA and RA have shown that synovial neutrophils express HLA-DR, a marker not commonly expressed by neutrophils, suggesting a role for neutrophils in inducing adaptive immunity (5, 6). We have previously shown that synovial neutrophils fail to suppress *T*-cell proliferation, a phenomenon believed to be partly driven by migration and impaired reactive oxygen species (ROS) production (7). Additionally, synovial neutrophils have impaired phagocytosis, suggesting compromised clearance and increased autoantigenic burden (4). Still, migration, as well as activation with SF, did not fully explain the synovial neutrophil phenotype (4, 7). Here, we instead investigated the contribution of S-Fib in driving neutrophil activation. Data on S-Fib from arthritis patients show that they are heterogenous and potent cytokine producers with altered surface marker expression and may thus influence neutrophil function in several ways (8, 20). Indeed, studies in RA show a clear interaction between S-Fib and neutrophils. For example, S-Fib can internalize neutrophil remnants and present antigens on MHC II, potentially promoting autoreactivity through activation of autoreactive lymphocytes (15). Still, evidence of the role of S-Fib in driving neutrophil activation in oJIA is lacking.

As expected, our data showed no effect of healthy S-Fib on the neutrophil phenotype. Moreover, our data only showed a mild influence of oJIA S-Fib on neutrophil phenotype and function. Thus, other factors than direct co-culture with S-Fib might drive the synovial neutrophil phenotype, such as migration, hypoxia and multi-factorial interactions. As previously mentioned, we have shown that migration induces alterations in healthy neutrophils, generating alterations reminiscent to that of synovial neutrophils. Hence, recruitment of neutrophils by S-Fib, and subsequent migration through the synovium, may explain several aspects of the neutrophil phenotype in synovial fluid. Still, S-Fib, with prior SF priming, prevented neutrophil apoptosis. Priming of S-Fib is a phenomenon believed to be important for the development of inflammatory S-Fib in the synovium (21). Indeed, S-Fib are sensitive to inflammatory stimuli and can mount stronger inflammatory responses upon activation (22, 23). Interestingly, data on synovial neutrophils from patients with different forms of arthritis suggest that their synovial phenotype is partly due to ageing (6). The prolonged survival observed could be crucial to allow the neutrophil to acquire its inflammatory features *in vivo*. A study on RA S-Fib found that S-Fib activated by IL-17 and TNF prolonged neutrophil survival, and that this was due to soluble factors, primarily GM-CSF (16). This is in contrast to our results, suggesting that cell-cell contact is required for the prolonged survival. However, this discrepancy could be due to multiple factors, such as the stimuli used (cytokines vs. SF) and disease parameters (RA vs. oJIA). Regardless, we collectively show that S-Fib from arthritis patients drive neutrophil survival in the inflamed joint and our data provide a possible mechanism to the generation of aged neutrophils in the synovium of oJIA patients. Aged neutrophils have several implications in the pathogenesis. For example, prolonged survival could suggest sustained detrimental effector functions, such as ROS production. Also, ageing could be crucial for the neutrophil phenotype and the expression of markers related to processes such as antigen presentation (6). In short, prolonged neutrophil survival could be detrimental in the pathogenesis of arthritis.

This study has some limitations. Firstly, ethical, and logistical limitations prevented us from obtaining biopsies to isolate S-Fib directly from tissue. S-Fib in the synovium are known to be diverse, exhibiting different functions depending on the subtype (20). Therefore, by isolating S-Fib solely from the SF, we may inadvertently focus on a specific subtype, potentially overlooking diverse influences of tissue-resident subtypes. However, the challenge of isolating S-Fib from SF is a common issue in JIA research, as most studies within this patient group resort to this method. Secondly, the absence of S-Fib from pediatric controls limits our ability to draw conclusions about whether the observed effects are specific to the disease mechanisms or could be attributed to S-Fib in general. Indeed, comparisons to disease controls, including other JIA subtypes, should be explored in future studies, to determine potential differences and similarities across diseases. Moreover, as we aimed to study the influence of S-Fib on neutrophils, we did not study the impact of neutrophils on the S-Fib. It is likely that this interaction is bilateral and represents an area that needs to be explored. Thirdly, all patients contributing S-Fib underwent NSAID treatment at the time of sampling. This factor could potentially influence the phenotype and function of S-Fib. Nevertheless, considering that the cells are isolated through passaging, we expect that any residual effects of the drugs and their influence would be minimal.

In conclusion, S-Fib from patients with oJIA recruit neutrophils and extend their lifespan through a mechanism dependent on cell-to-cell contact. This process may play a crucial role in the accumulation of neutrophils within the synovium and thus their contribution to the pathogenesis. Still, the impact of S-Fib on the phenotype and function of neutrophils appears to be limited. This observation implies that alternative mechanisms, such as migration, are likely responsible for triggering the activation of neutrophils within the joint.

## Data Availability

The raw data supporting the conclusions of this article will be made available by the authors, without undue reservation.
